# Effect of simvastatin on the SIRT2/NF-κB pathway in rats with acute pulmonary embolism

**DOI:** 10.1080/13880209.2018.1508239

**Published:** 2018-12-03

**Authors:** Zhi-Yao Wu, Hui Li, Yong-Jun Tang

**Affiliations:** Department of Respiratory Medicine (Department of Respiratory and Critical Care Medicine), National Key Clinical Specialty, Xiangya Hospital, Central South University, Changsha, China

**Keywords:** Inflammatory cytokines, MMPs, pulmonary artery pressure

## Abstract

**Context:** Statins have been widely used in acute pulmonary embolism (APE), while simvastatin has been well-established for the prevention of pulmonary hypertension, which was supposed to be an attractive recommendation for APE treatment.

**Objective:** The current article studies the effect of simvastatin on the SIRT2/NF-κB pathway in rats with APE.

**Materials and methods:** Sprague-Dawley rats were divided into four groups (*n* = 24 per group): control group, rats were treated with saline once daily for 14 days before administration of saline (sham group) or a suspension of autologous emboli (APE group), or rats were treated with simvastatin (10 mg/kg) for 14 days before administration of autologous emboli (APE + simvastatin) group. The RVSP, mPAP and the arterial blood gas was analyzed. Besides, plasma inflammatory cytokines and MMPs levels, as well as the expression of SIRT2/NF-κB pathway were determined.

**Results:** Compared with the control and sham groups, the levels of mPAP (31.06 ± 3.47 mmHg), RVSP (35.12 ± 6.02 mmHg), A-aDO_2_ (33.14 ± 6.16 mmHg) and MMP-9 (6.89 ± 0.84 ng/mL) activity were significantly elevated, but PaO_2_ (66.87 ± 7.85 mmHg) was highly decreased in rats from APE group at 24 h after APE. Meanwhile, the inflammatory changes were aggravated by the enhanced levels of TNF-α (138.85 ± 22.69 pg/mL), IL-1β (128.47 ± 22.14 pg/mL), IL-6 (103.16 ± 13.58 pg/mL) and IL-8 (179.28 ± 25.79 pg/mL), as well as increased NF-κB (5.29 ± 0.47 fold), but reduced SIRT2 (59 ± 6% reduction), and eNOS (61 ± 5% reduction) mRNA in APE rats. APE rats treated with simvastatin led to a significant opposite trend of the above indexes.

**Conclusions:** Simvastatin protects against APE-induced pulmonary artery pressure, hypoxemia and inflammatory changes probably due to the regulation of SIRT2/NF-κB signalling pathway, which suggest that simvastatin may have promising protective effects in patients with APE.

## Introduction

Acute pulmonary embolism (APE) is one of the most prevalent acute cardiovascular diseases, following myocardial infarction and stroke, with the characteristics of high incidence and mortality rate, high misdiagnosis rate, but low diagnosis rate, seriously threatening the safety and life quality of patients (Burns and Haramati 2012). As reported, there was about 8% of pulmonary embolism (PE) patients who are diagnosed and treated in time, while untreated PE related to a mortality number ∼30% (Toba et al. 2010). Generally, the pulmonary artery hypertension (PAH) induced by the sharp rise of pulmonary vascular resistance can lead to the rapid increase of pressure load on the right ventricle (RV), thus causing acute right ventricular failure and circulatory shock, which is the major cause of death by APE (Venkatesh 2011; Cau et al. 2013). In recent decades, evidence suggested that a number of drugs, such as l-arginine (Souza-Costa et al. 2005), sildenafil (Dias-Junior et al. 2009), nitric oxide_2_ (Dias-Junior et al. 2010) and endothelin receptor antagonists, had been used to attenuate APE-induced increases in pulmonary hypertension and reduce APE-related mortality, but the effects were not entirely satisfactory due to the obvious elevation in pulmonary vascular tone and the deterioration in gas exchange. Therefore, it is of great significance to find new strategies for APE treatments.

Statins, the so-called inhibitors of 3-hydroxy-3-methyl glutaryl coenzyme A reductase (Katsakiori et al. 2011), are considered as novel cholesterol-lowering agents, and exert pleiotropic immune-modulatory properties in improving endothelial function, alleviating inflammation, reducing oxidative stress, enhancing fibrinolytic activity and preventing thrombosis (Girotra et al. 2012; Owens et al. 2014). In recent years, various statins, including simvastatin, with distinct pharmacologic properties, had been reported to effectively mitigate the risk of venous thromboembolism (Vte) (Patterson et al. 2013; Feng et al. 2016) and alleviate the neurological impairment caused by ischemic stroke (Shabanzadeh et al. 2005), and it was worthy to mention that statins treatment might be an attractive recommendation for the long-term treatment of PE (Ray 2013; Limbrey and Howard 2015).

Sirtuin-2 (SIRT2), a member of the silent information regulator (Sirtuin) family, participates in many pathological and physiological processes, including DNA regulation, metabolism, cell-cycle, inflammation, tumorigenesis, and especially in the regulation of lifespan (Wang et al. 2007; Matsushima and Sadoshima 2015). As revealed by Kilic et al. (2015), treatment with statins can inhibit the expression of SIRT1, which is highly homologous to SIRT2, to protect the cardiovascular system of patients with coronary artery diseases (CAD). In the study by Zhang et al. (2015), simvastatin could down-regulate TNF-α/NF-κB signalling pathway to blunt retinal cell apoptosis induced by ischemia-reperfusion. More importantly, SIRT2 can involve in the regulation of NF-κB signalling pathway via the deacetylation of p65Lys310, as indicated by Rothgiesser et al. (2010). And the activation of NF-κB pathway can affect the role of C-reactive protein (CRP) in regulating pulmonary arterial endothelial cells in chronic thromboembolic pulmonary hypertension (Wynants et al. 2013). Taking this information into account, we constructed a model of APE in rats by injecting autologous thrombus *in vitro*, to investigate whether simvastatin could play a protective role in APE via the regulation of the SIRT2/NF-κB pathway.

## Materials and methods

### Ethics statement

This study was approved by the Ethics Committee for Laboratory Animals in Xiangya Hospital, Central South University. All experimental procedures and animal treatments were strictly in accordance with the *Guide for the Care and Use of Laboratory Animals* published by the US National Institutes of Health (NIH Publication No. 85-23) (National Institutes of Health 1996).

### Establishment and grouping of APE rat models

**Figure 1.**
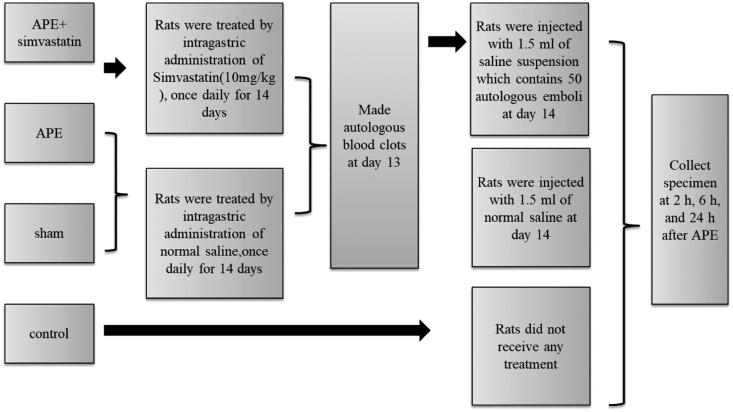
Animal grouping and experimental procedures.

Ten-week-old male Sprague-Dawley rats of SPF grade (weighing 340–360 g; Silaike Co, Shanghai, China) had food and water freely available on normal circadian rhythm in a clean animal room at 22–25 °C and were maintained for experiments after 4–5 days. These rats were randomly divided into four groups (*n* = 24 per group): control group, sham operation group (sham), APE model group (APE) and APE + simvastatin (10 mg/kg simvastatin; Hangzhou MSD Pharmaceutical Co. Ltd) group (Lin et al. 2015). The rats with anaesthetic accidents or died halfway were excluded from the study. The injection of autologous thrombus was used to construct APE rat models (Diaz et al. 2012). To be specific, rats were anaesthetized by intraperitoneal injection of 10% chloral hydrate and fixed in the supine position. A micro catheter with the diameter of 1.1 mm was inserted into the left femoral artery to take 0.5 mL of arterial blood. Next, the microcatheter was kept at room temperature for 30 min and heated for 5 min in the warm water of 60 °C. Then, the thrombus in the catheter was pushed into a sterile Petri dish, cut into embolus of 1.1 × 4 mm, and preserved in a refrigerator at 4 °C. Rats in each group were divided into three-time groups, namely 2, 6 and 24 h, with 8 rats at each time point. Animal grouping and experiment procedures are shown in Figure 1.

### Measurements of right ventricular systolic pressure (RVSP) and mean pulmonary arterial pressure (mPAP)

A polystyrene microcatheter (inner diameter 0.9 mm) was prefilled with heparin saline and connected to the BL-420E biological function experiment system (Chengdu TME Technology Co, Ltd., China), and zero calibration was implemented. The rats were anaesthetized to separate the right external jugular vein, the cannula was inserted into the pulmonary artery through the right ventricle and a tight ligature was placed around the main trunk of pulmonary artery. When the typical curve of right ventricular pressure appeared for a less while, the RVSP was recorded. Mean pulmonary arterial pressure was measured from the side of the fluid intubation with pulmonary arterial intubation pressure transducers (COBE, Arvada, CO).

### Specimen collection

After the measurement of mPAP and RVSP, the right common carotid artery (RCCA) was separated to extract about 1 mL of arterial blood. Next, the PaO_2_, PaCO_2_, PH, and A-aDO_2_ were determined. The arterial blood of each group was placed in EDTA anticoagulant tubes and centrifuged for 10 min at 4 °C at the rate of 3000 rpm to separate plasma, which was preserved in sterile EP (Eppendorf) tubes in a refrigerator at −80 °C. The chest of rats was cut open and injected with 60 mL of cold saline. When the lung became white, the right pulmonary hilum was ligated, and the right lung was cut and stored in liquid nitrogen. Next, the skin of the neck was cut open to expose the trachea, and 6 mL 4% paraformaldehyde was injected to make the left lung swell. Then, the left pulmonary hilum was ligated, and the left lung was cut and fixed for 48 h in 4% paraformaldehyde for later pathological examinations. The hearts were rapidly removed, right ventricular (RV) samples were collected and washed in cold saline, snap frozen and stored at −80 °C.

### Pathological examination of lung tissues by HE staining

The left lung fixed in paraformaldehyde was taken for dehydration with gradient alcohol, embedded with paraffin (Thermo) and cut into serial lung sections of 5 μm in thickness. After conventional dewaxing, sections were stained with hematoxylin-eosin (HE, Wuhan Boster Biological Technology Ltd., Wuhan, Hubei, China), 3 min in hematoxylin and 3 min in eosin. Next, the sections were mounted and observed under a microscope (CX31, Olympus Optical Co., Ltd, Tokyo, Japan) for the pathological changes in lung tissues of rats in each group.

### Detection of inflammatory cytokines by enzyme-linked immunosorbent assay (ELISA)

In strict accordance with the instructions on the ELISA Kit (Beijing Neobioscience Biotechnology Co. Ltd.), the expressions of tumor necrosis factor α (TNF-α), interleukin-1β (IL-1β), interleukin-6 (IL-6) and interleukin 8 (IL-8), as well as matrix metalloproteinase (MMP)-2 and MMP-9, was detected, and the results were analyzed. The experiment was repeated three times to calculate the mean valve.

### Measurement of MMPs in RV by SDS-PAGE gelatin zymography and *in situ* zymography

To assess MMP-2 and MMP-9 in the RV, gelatin zymography was performed as previously described (Cau et al. 2013). Briefly, RV samples were subjected to electrophoresis on SDS PAGE co-polymerized with gelatin (1%) as the substrate. Gelatinolytic activities were normalized with regard to an internal standard (fetal bovine serum) to allow gelatin analysis and comparison. Enzyme activity was assayed by densitometry using ImageJ Program. MMP-2 and MMP-9 were identified as bands at 72 and 92 kD, respectively. Next, *in situ* MMPs activity was measured in frozen RV tissue using dye-quenched (DQ)–gelatin (Molecular Probes, Oregon, USA) as a fluorogenic substrate, according to the previous studies (Neto-Neves et al. 2011; Sousa-Santos et al. 2012).

### Quantitative real-time polymerase chain reaction (qRT-PCR)

The lung tissue of rats was ground with a tissue homogenate machine (Thermo), and the total RNA was extracted with a TRIZOL reagent (Invtrogen). Based on the gene sequences available in the Genbank database, the primers (Table 1) for PCR were designed with the software Primer 5.0 and synthesized by Shanghai GenePharma Co., Ltd. ABI PRISM 7500 real-time PCR System (ABI) was used with GAPDH as the internal reference gene. The relative expression level of target genes was calculated using 2^−ΔΔ^Ct method: ΔCt = CT_(target gene)_ − CT_(internal reference gene)_; and ΔΔCt = ΔCt_(treatment group)_ − ΔCt_(control group)_. The experiments were repeated three times to get the mean value.

**Table 1.** Primers for qRT-PCR.

Gene	Sequence
SIRT2	
Forward primer	5'- TACCCAGAGGCCATCTTTGA -3'
Reverse primer	5'- TGATGTGTGAAGGTGCCGT -3'
NF-κB	
Forward primer	5'- GTGCAGAAAGAAGACATTGA -3'
Reverse primer	5'- AGGCTAGGGTCAGCGTATGG -3'
GAPDH	
Forward primer	5′- TCAAGAAGGTGGTGAAGCAG -3′
Reverse primer	5′- AGGTGGAAGAATGGGAGTTG -3′

## Western blot

The lung tissue of rats was ground with a tissue homogenate machine (Thermo) to extract the total protein, which was determined for concentration by following the instruction on the BCA (Bicinchoninic acid) kit (Wuhan Boster Biological Technology Ltd., Wuhan, Hubei, China). Next, the protein sample was added with loading buffer and boiled for 10 min at 95 °C before loading the sample by 40 μg/well. Then, electrophoresis was applied to isolate proteins with 10% polyacrylamide gel. The proteins on the PVDF (polyvinylidene fluoride) transfer membrane was closed for 1 h with 5% BSA at room temperature before adding primary antibodies for overnight incubation at 4 °C, including NF-κB (1:100 dilution, Santa Cruz Biotechnology, Inc., Santa Cruz, CA, USA), SIRT2 (1:50 dilution, Abcam Inc., Cambridge, MA, USA), endothelial nitric oxide synthase (eNOS) (1:1000 dilution, Abcam Inc., Cambridge, MA, USA) and β-actin (1:1000 dilution, Abcam Inc., Cambridge, MA, USA). Then, after rinsing with TBST (Tris-buffered saline Tween-20) 5 min ×3 times, corresponding secondary antibodies were added for 1 h of incubation. After another round of rinse with TBST 5 min ×3 times, chemiluminescence reagent was used for developing. The internal reference gene was β-actin and the experiment was repeated three times.

### Statistical method

The statistical software SPSS18.0 was used to analyze the data. Two groups of measurement data that obey normal distribution were compared using Student’s *t*-test, while the comparison among multiple groups was analyzed using One-Way ANOVA. *p <* 0.05 meant the difference was of statistical significance.

## Results

### Effect of simvastatin on the arterial blood gas analysis, mPAP and RVSP in APE rats

**Figure 2.**
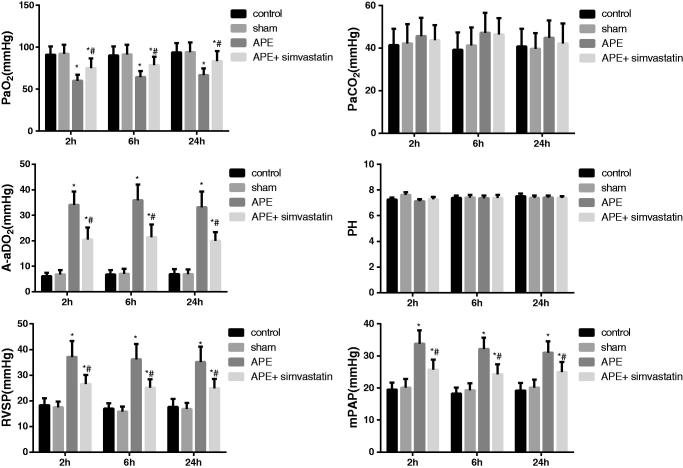
Effect of simvastatin on the arterial blood gas analysis, mPAP and RVSP at different time points in each group. *p < 0.05 compared with the Control group; #*p* < 0.05 compared with the APE group.

As shown in Figure 2, there was no significant difference between the control group and the sham group in all indexes about 2, 6 and 24 h after APE (all *p* > 0.05). However, in comparison with the two groups, the rats in the APE group were significantly higher in mPAP, RVSP and A-aDO_2_ level but statistically lower in PaO_2_ level at each different time point (all *p* < 0.05). On the contrary, APE rats treated with simvastatin showed a significant decrease in mPAP, RVSP and A-aDO_2_ level and the obvious increase in PaO_2_ level at different time points (all *p* < 0.05). Moreover, no significant difference was found in PaCO_2_ and PH value among groups at different time points (all *p* > 0.05).

### Pathological changes of lung tissues in APE rats

**Figure 3.**
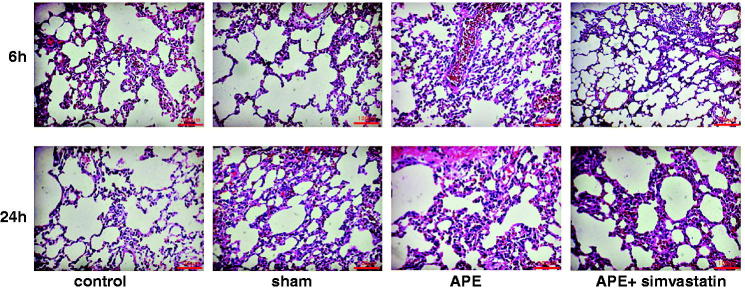
Pathological changes in lung tissues in each group after 6 h and 24 h of APE (×200).

According to the HE staining, rats in the control group and sham group did not show thrombus in all levels of pulmonary artery about 6 and 24 h, and the alveolar structure was preserved with clear alveolar space, without inflammatory cell infiltration in the inter-alveolar septa, and no evident bleeding and exudate. However, rats in the APE group had several thrombi in the middle and small pulmonary arteries and infiltration of many neutrophile granulocytes and macrophage into the pulmonary arterial wall; furthermore, the alveolar septa became widened with the infiltration of inflammatory cells; and many liquid and red cells exuded from the alveolus. Moreover, the pathological changes in lung tissues of rats in the APE + simvastatin group were similar to those rats in the APE group, but the inflammation was obviously alleviated in their pulmonary arterial wall and lung tissues (Figure 3).

### Effect of simvastatin on expressions of inflammatory cytokines in APE rats

**Figure 4.**
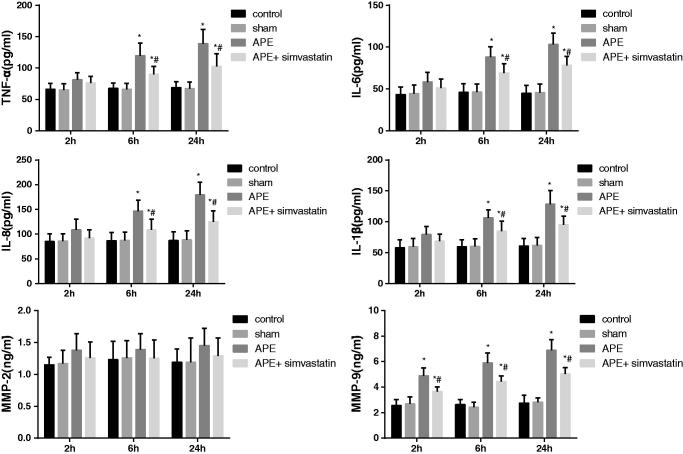
Effect of simvastatin on expressions of inflammatory cytokines detected by ELISA in each group. *p < 0.05 compared with the Control group; #*p* < 0.05 compared with the APE group.

ELISA method was used to detect the expression of inflammatory cytokines in the arterial plasma of rats at different time points. As demonstrated in Figure 4, no significant difference was observed regarding the expressions of inflammatory cytokines between the control and sham groups at each time point (all *p* > 0.05). But, the expression levels of TNF-α, IL-1β, IL-6 and IL-8 were detected to be gradually elevated in APE rats, which were of statistical significance after 6 and 24 h APE (all *p* < 0.05). Conversely, when APE rats with the treatment of simvastatin, the expression of TNF-α, IL-1β, IL-6 and IL-8 exhibited an opposite consequence (all *p* < 0.05). Besides, the up-regulated MMP-9 was displayed in rats after 2, 6 and 24 h of APE (*p* < 0.05) and was significantly reduced after treatment with simvastatin (*p* < 0.05). No significant changes were found in MMP-2 (*p* > 0.05).

### Effect of simvastatin on RV MMP activity in APE rats

**Figure 5.**
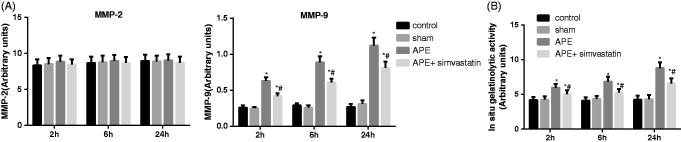
Effect of simvastatin on RV MMP activity in APE rats. (A) Activity of MMP-2 and MMP-9 were determined by SDS-PAGE gelatin zymography; (B) Quantification of *in situ* gelatinolytic activity of right ventricle samples; *p < 0.05 compared with the Control group; #*p* < 0.05 compared with the APE group.

To determine changes in RV MMP activity induced by APE, gelatin and *in situ* zymography were performed. As shown in the representative zymogram in Figure 5(A), no significant difference was observed regarding the RV MMP-2 activity among each group at each time point (all *p* > 0.05); however, significant higher activity of MMP-9 was found in RV samples after 2, 6 and 24 h of APE (all *p* < 0.05). The *in situ* gelatinolytic activity increased in rats at 6 and 24 h after APE (Figure 5(B), *p* < 0.05). Importantly, rats in the APE + simvastatin group were lower in RV MMP-9 activity and RV gelatinolytic activity than rats in the APE group at 2, 6 and 24 h, respectively (all *p* < 0.05).

### Effect of simvastatin on the SIRT2/NF-κB pathway in APE rats

**Figure 6.**
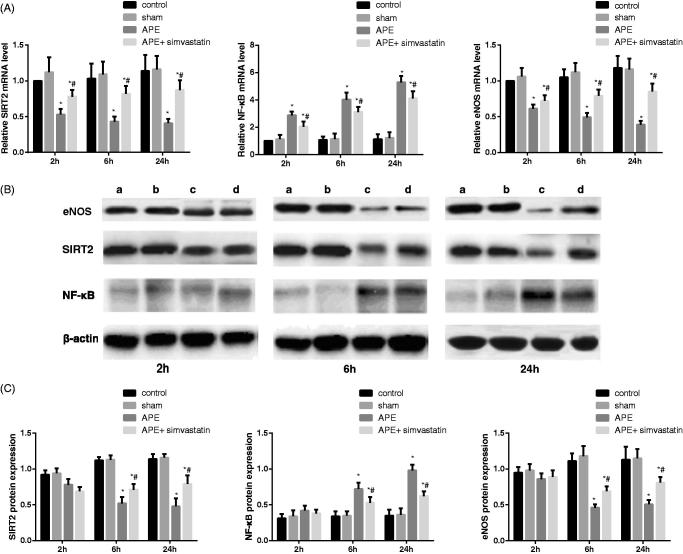
Effect of simvastatin on the SIRT2/NF-κB pathway detected by qRT-PCR and Western Blot in each group. (A) The relative mRNA levels of SIRT2/NF-κB in each group at different time points detected by qRT-PCR; (B) The protein expression of SIRT2/NF-κB in each group at different time points detected by Western Blot; a: Control group; b: Sham group; c: APE group; d: APE + simvastatin group; (C) Comparison of the protein expression of SIRT2/NF-κB in each group at different time points; *p < 0.05 compared with the Control group; #*p* < 0.05 compared with the APE group.

The results of qRT-PCR and Western Blot are shown in Figure 6. Rats in the control group showed no significant difference from rats in the sham group regarding the mRNA level and protein expression of eNOS and SIRT2/NF-κB pathway at each time point (both *p* > 0.05). The decreased SIRT2 and eNOS mRNA and the increased NF-κB mRNA were discovered after 2, 6 and 24 h of APE; and meanwhile, the significant reduction of eNOS and SIRT2 protein and the obvious elevation of NF-κB protein appeared in rats at 6 and 24 h after APE (all *p* < 0.05). Of note, rats in the APE + simvastatin group were appreciably higher in the expression of eNOS and SIRT2 mRNA and evidently lower in the NF-κB mRNA level than rats in the APE group at 2, 6 and 24 h respectively, which were especially significant at 6 and 24 h (all *p* < 0.05).

## Discussion

Pulmonary hypertension and hypoxemia are the major pathophysiological manifestations of APE. To be specific, owing to the obstruction of pulmonary arteries with embolization, the imbalance of ventilation/perfusion induced by the improper redistribution of blood flow would result in hypoxemia, which was mainly reflected by the reduction of PaO_2_ and the increase of A-aDO_2_ (Hsu et al. 2006; Toba et al. 2010). However, the blood flow was restricted by the thrombus with the increase of pulmonary vascular resistance and mPAP, as well as the elevation of RVSP and ultimately leading to right ventricular failure or even death (Gutte 2011). In this work, the increased mPAP, RVSP and A-aDO_2_ and the decreased PaO_2_ were indeed presented in APE rats. However, the APE after treated with simvastatin, the pulmonary vascular resistance and mPAP, as well as the right ventricle afterload was alleviated, thereby improving the pulmonary hemodynamics and hypoxemia in APE rats (Zagorski et al. 2010; Zhao et al. 2015). In addition, multiple thrombi were observed in the middle and small pulmonary arteries of APE rats, and there were more neutrophils and macrophages infiltrating the pulmonary artery wall, which was similar to the results in a previous study (Zhou et al. 2017). Not surprisingly, APE rats pretreated with simvastatin in our study have obviously alleviated the inflammation in the pulmonary artery wall and lung tissues. There was evidence that statins, such as pravastatin and fluvastatin, can significantly attenuate the adhesion via inhibition of the adhesion molecules expression to improve vascular inflammatory reaction (Omi et al. 2003), which suggested the inhibitory effect of simvastatin on the APE-induced local inflammatory response possibly through the inhibition of neutrophil adhesion, aggregation and activation. Furthermore, APE has been demonstrated to be associated with increased MMP-9 and enzymatic activity in the RV, and treatment with simvastatin blunted APE-induced dgelatinolytic activity. According to a previous study, the inhibition of MMP may reduce APE-induced pulmonary hypertension to some extent (Lualdi and Goldhaber 1995). Also, the abrupt increase in pulmonary vascular resistance during APE resulted in the increased RV afterload, further leading to myocardial ischemia and RV dysfunction. Thus, it is possible that part of the cardiac protective effects of simvastatin would be attributed to the inhibition of the modulation of pulmonary vascular tone by MMPs (Chow et al. 2007).

In addition, the significant decrease of SIRT2 and eNOS and the markedly increase of NF-κB were detected at the mRNA level and protein expression in rats after APE. Similar to other members of the Sirtuin family, SIRT2 is a highly conserved and NAD+ (nicotinamide adenine dinucleotide)-dependent deacetylase which plays a vital role in various biological processes (Donmez and Outeiro 2013). In addition, NF-κB was credited as a widely-distributed inducible transcription factor, participated in the modulation of many genes in a variety of cellular processes, including apoptosis, cell survival, immune response and so on (Chen et al. 2014). Furthermore, the activated NF-κB could cause the release of inflammatory cytokines, including TNF-α, IL-1β, IL-6 and MMPs, and promote neutrophil accumulation, thereby aggravating inflammatory responses (Azzolina et al. 2003; Shi et al. 2014). Yang et al. (2007) reported that cigarette smoke exposure to lung macrophages decreased levels of SIRT1 and SIRT2, which was responsible for neutrophil influx, to be relevant to the increased activation of various NF-κB-dependent pro-inflammatory mediators. Also, the SIRT2 inhibitor, AK-7, can up-regulate its target gene by increasing the acetylation and nuclear translocation of NF-κB, thereby leading to the up-regulation of MMP-9 and the release of pro-inflammatory cytokines, which eventually aggravates the traumatic brain injury (Yuan et al. 2016). In addition, eNOS, an important enzyme in the production of NO, is present in vascular endothelial cells, and a decreased expression level or abnormality of eNOS protein has been shown in pulmonary hypertension (Cau et al. 2013). Greco et al. (2011) reported that inhibiting the expression of eNOS was associated with enhanced NF-κB activation and a significant increase in infarct volume in a cerebral ischemia/reperfusion rat model. Bao et al. (2018) also discovered that eNOS upregulation, at least in part, contributed to the protective effect of simvastatin against HBO-induced acute lung injury. In our study, with the simvastatin treatment, SIRT2 and eNOS have enhanced appreciably while NF-κB was reduced significantly, showing that simvastatin can increase the expression of SIRT2 and eNOS to exert a protective function, thereby inhibiting the expression of NF-κB to affect the expression of downstream inflammatory cytokines.

By detecting inflammatory cytokines via ELISA, we found an elevation of TNF-α, IL-1β, IL-6 and IL-8 in the plasma of rats at 6 h after APE, which was consistent with a previous study (Liu et al. 2007). Yokota et al. (2006) indicated a beneficial role of simvastatin patients with rheumatoid arthritis, which could significantly inhibit the secretion of IL-6 and IL-8 and reduce the proliferation of fibroblast-like synoviocytes induced by TNF-α. Also, simvastatin could decrease the pro-inflammatory, thrombotic and pro-aggregation effects of IL-17 on IL-6 mRNA, and thus restoring the platelet aggregation of endothelial cells to a normal level (Hot et al. 2013). Our study also found the obvious increase of MMP-9 in the rats’ plasma after 2 h of APE. Previous clinical evidence stating that the expression increased level of MMP-9 was positively related to the pulmonary vascular resistance and pulmonary arterial hypertension in APE (Uzuelli et al. 2008), due to the release of local cytokines and various inflammatory cell infiltration caused by thromboembolism (Fortuna et al. 2007). More importantly, many experiments have demonstrated that MMPs inhibitors or inhibition the activity of MMPs can modulate vascular contractility to reduce pulmonary arterial hypertension and pulmonary vascular resistance (Souza-Costa et al. 2007; Dias-Junior et al. 2009). Souza-Costa et al. (2007) pointed out that pretreatment of APE rats with atorvastatin could improve the 24 h survival rate and alleviate the pulmonary hypertension induced by APE through reducing the expression of MMP-9. Similarly, the remarkable decrease of inflammatory cytokines and MMP-9 were exhibited in APE rats with the treatment of simvastatin, implying that simvastatin has an anti-inflammatory effect in APE rats, via improving inflammatory cell infiltration and reducing the secretion of inflammatory cytokines, as well as down-regulation of MMP-9. More importantly, Biererafi et al. (2015) performed a case-control study and found that statins may be an interesting option for long-term secondary prevention in patients with PE, which suggested that simvastatin may also have promising protective effects in patients with APE. Therefore, a clinical trial would be an important next step in our future studies to validate the experimental evidence provided here and in other recent studies.

In summary, our study indicated that APE rats treated with simvastatin can effectively reduce mPAP and RVSP, alleviate hypoxemia and inflammation, via enhancement of SIRT2 and inhibition of NF-κB, thus providing a new idea for the clinical treatment of APE.
